# Case Report: Key Role of the Impella Device to Achieve Complete Revascularization in a Patient With Complex Multivessel Disease and Severely Depressed Left Ventricular Function

**DOI:** 10.3389/fcvm.2021.784912

**Published:** 2021-12-02

**Authors:** Giovanni Monizzi, Luca Grancini, Paolo Olivares, Antonio L. Bartorelli

**Affiliations:** ^1^Centro Cardiologico Monzino, IRCCS, Milan, Italy; ^2^Department of Biomedical and Clinical Sciences “Luigi Sacco”, University of Milan, Milan, Italy

**Keywords:** coronary intervention, PCI, Impella, LV assistance, ventricular fibrillation

## Abstract

**Background:** Left ventricle (LV) assist devices may be required to stabilize hemodynamic status during complex, high-risk, and indicated procedures (CHIP). We present a case in which elective hemodynamic support with the Impella CP device was essential to achieve complete revascularization with PCI in a patient with complex multivessel disease and severely depressed LV function.

**Case Summary:** A 45-year-old male with no previous history of cardiovascular disease presented to the emergency department for new onset exertional dyspnoea. Echocardiography showed severely depressed LV function (EF 27%) that was confirmed with cardiac magnetic resonance. Two chronic total occlusions (CTOs) of the proximal right coronary artery (RCA) and left circumflex coronary artery (LCx) were found at coronary angiography. After Heart Team evaluation, PCI with Impella hemodynamic support was planned. After crossing and predilating the CTO of the LCx, ventricular fibrillation (VF) occurred. No direct current (DC) shock was performed because the patient was conscious thanks to the support provided by the Impella pump. About 1 min later, spontaneous termination of VF occurred. Afterwards, the two CTOs were successfully treated with good result and no complications. Recovery of LV function was observed at discharge. At 9 months, the patient had no symptoms and echocardiography showed an EF of 60%.

**Discussion:** In this complex high-risk patient, hemodynamic support was essential to allow successful PCI. It is remarkable that the patient remained conscious and hemodynamically stable during VF that spontaneously terminated after 1 min, likely because the Impella pump provided preserved coronary perfusion and LV unloading. This case confirms the pivotal role of Impella in supporting CHIP, particularly in patients with multivessel disease and depressed LV function.

## Introduction

A percutaneous coronary intervention (PCI) performed in complex coronary anatomy, such as multivessel disease, left main involvement, chronic total occlusions (CTOs) and last remaining vessel, particularly in patients with poor left ventricle (LV) function is dubbed “CHIP” (complex, high-risk, and indicated procedure) (1). To deal with CHIP, mechanical circulatory support may be necessary to increase procedural safety and success (2). Several studies have demonstrated that the Impella pump (Abiomed, Danvers, MA, USA), a catheter-based miniaturized ventricular assist device, is safe, easy to implant and provides excellent hemodynamic support during high-risk PCI (3–6). The efficacy of this device has been proven superior to intra-aortic balloon pump (IABP) in this scenario (7). We present a case in which elective hemodynamic support with the Impella pump was essential for allowing complete revascularization in a young patient with complex multivessel coronary disease and severely depressed LV function.

## Case Presentation

**Figure 1:**
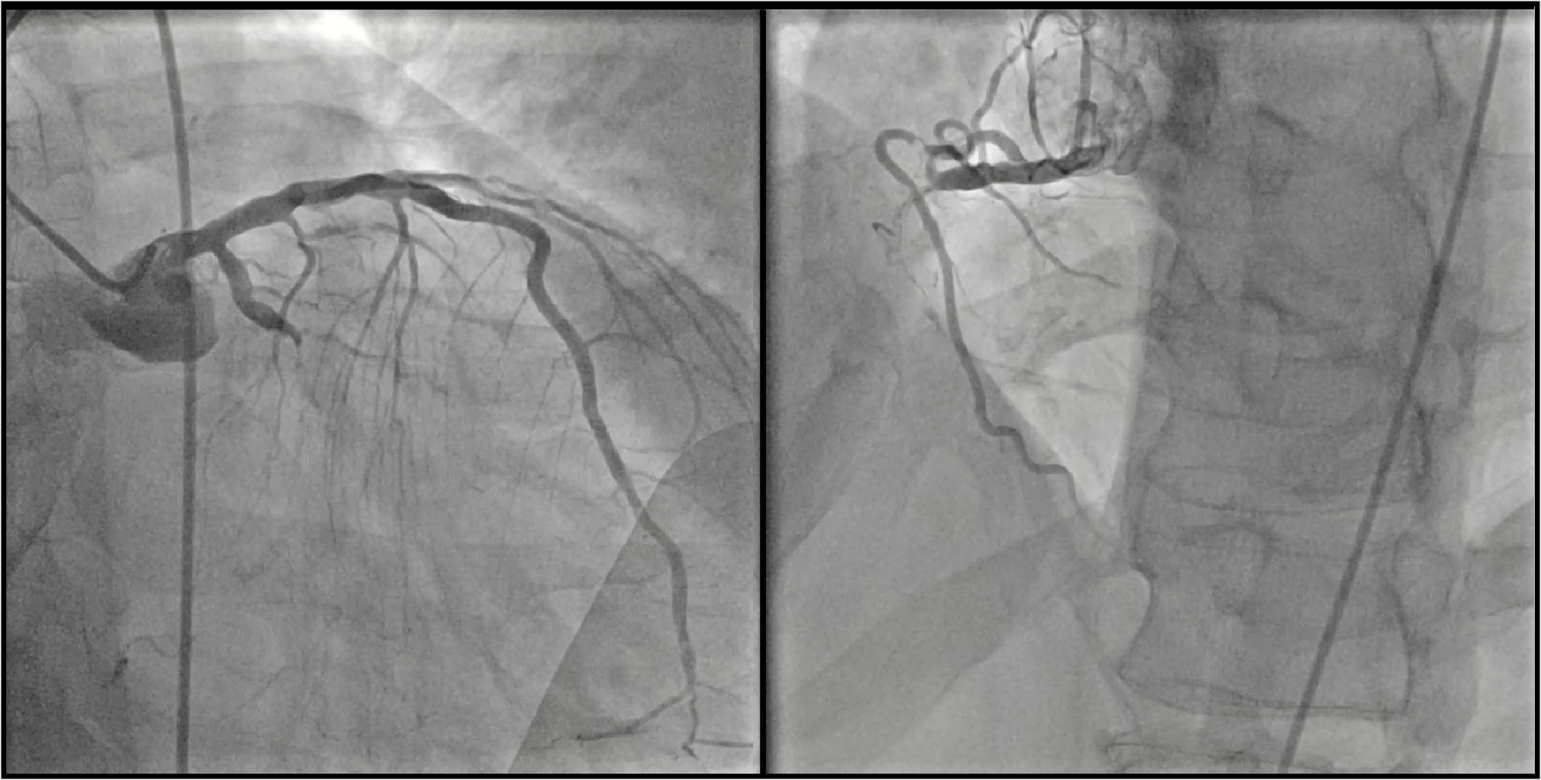
Coronary angiography. *Left panel*: chronic total occlusion of the mid left circumflex coronary artery. *Right panel*: chronic total occlusion of the proximal right coronary artery.

**Figure 2:**
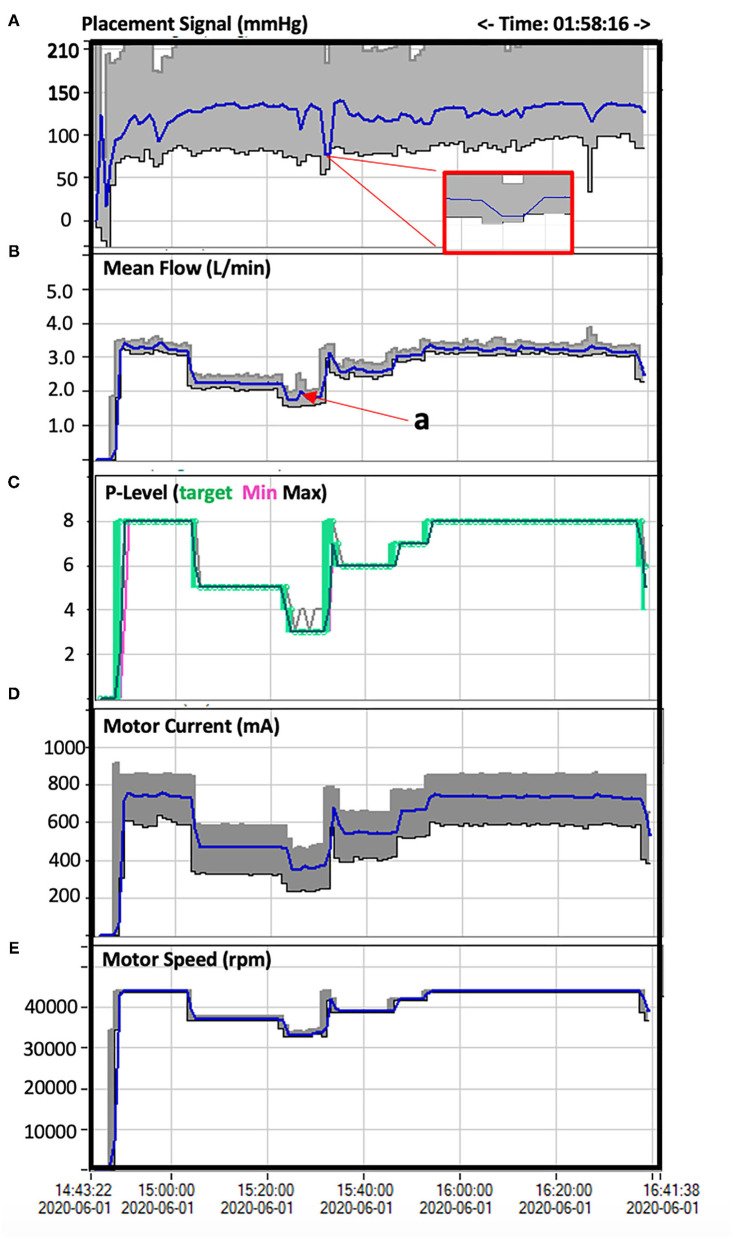
Impella CP parameters during the procedure. **(A)** the Placement Signal is an approximation of the central aortic pressure (mmHg) with the gray area representing the systolic and diastolic pressures and the blue line the mean aortic pressure. The red box highlights in magnified form the 1-min pressure drop and reduced pulsatility during the VF episode and the immediate increase of pressure and pulsatility after spontaneous termination of the tachyarrhythmia. **(B)** This curve represents the mean Impella flow (L/min) during the procedure. A short flow peak (**a**) is visible at maximal compromised cardiac function during VF due to proportional reversed response to compensate for the reduced cardiac flow. Impella flow is managed based on the Performance (P-Level) **(C)**, which corresponds to Motor Current (mA) **(D)** and motor speed (rpm) **(E)** and can be manually adjusted. High flow requires high Motor Current and Motor Speed (8). During the VF episode, Impella Performance and Flow were manually and gradually reduced (from P8 down to P3) in anticipation to DC shock and were increased again as soon as the patient stabilized after VF termination.

**Figure 3:**
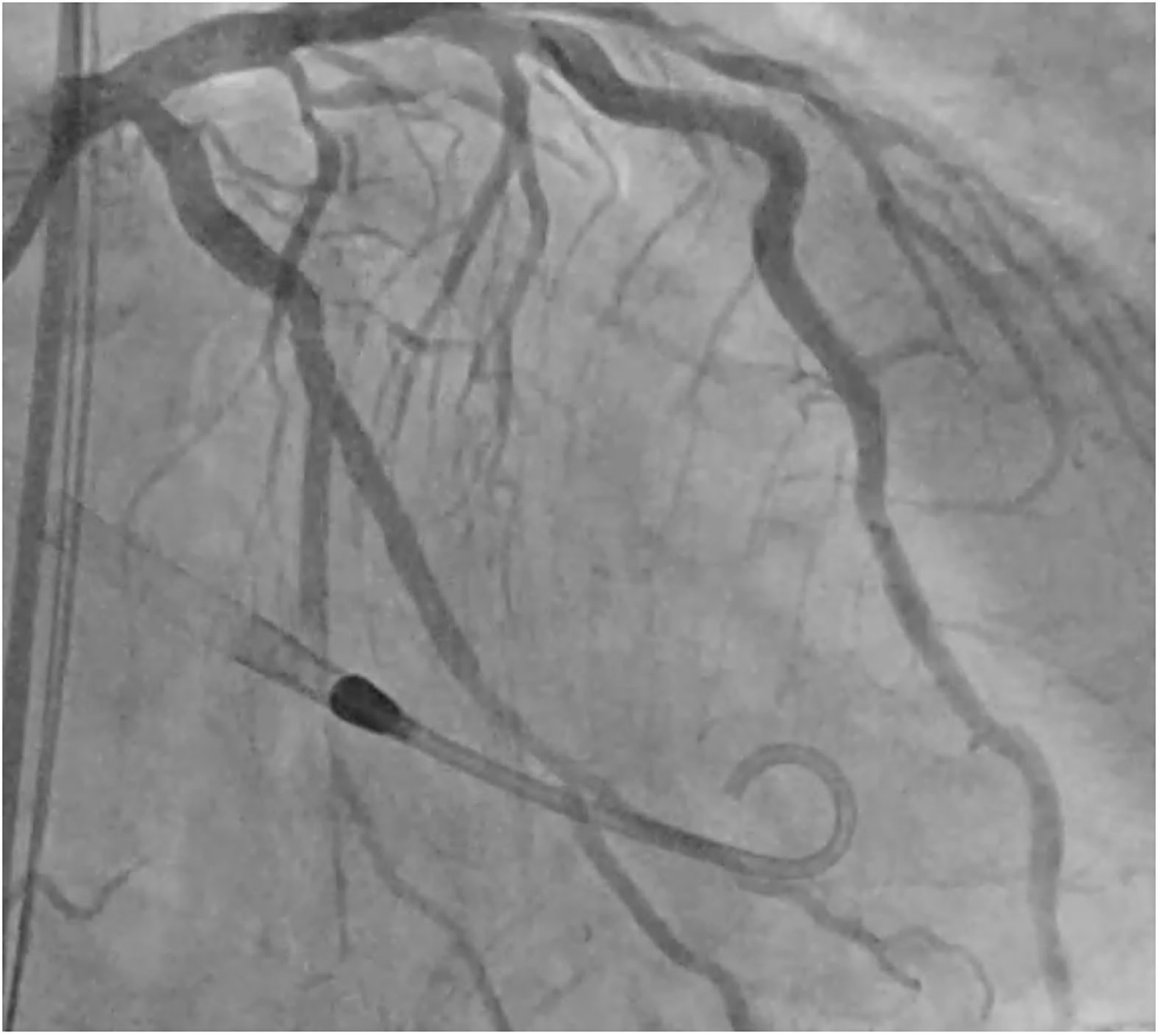
Left circumflex coronary artery after PCI. Final result after implantation of two drug-eluting stents using the T-stent technique.

**Figure 4:**
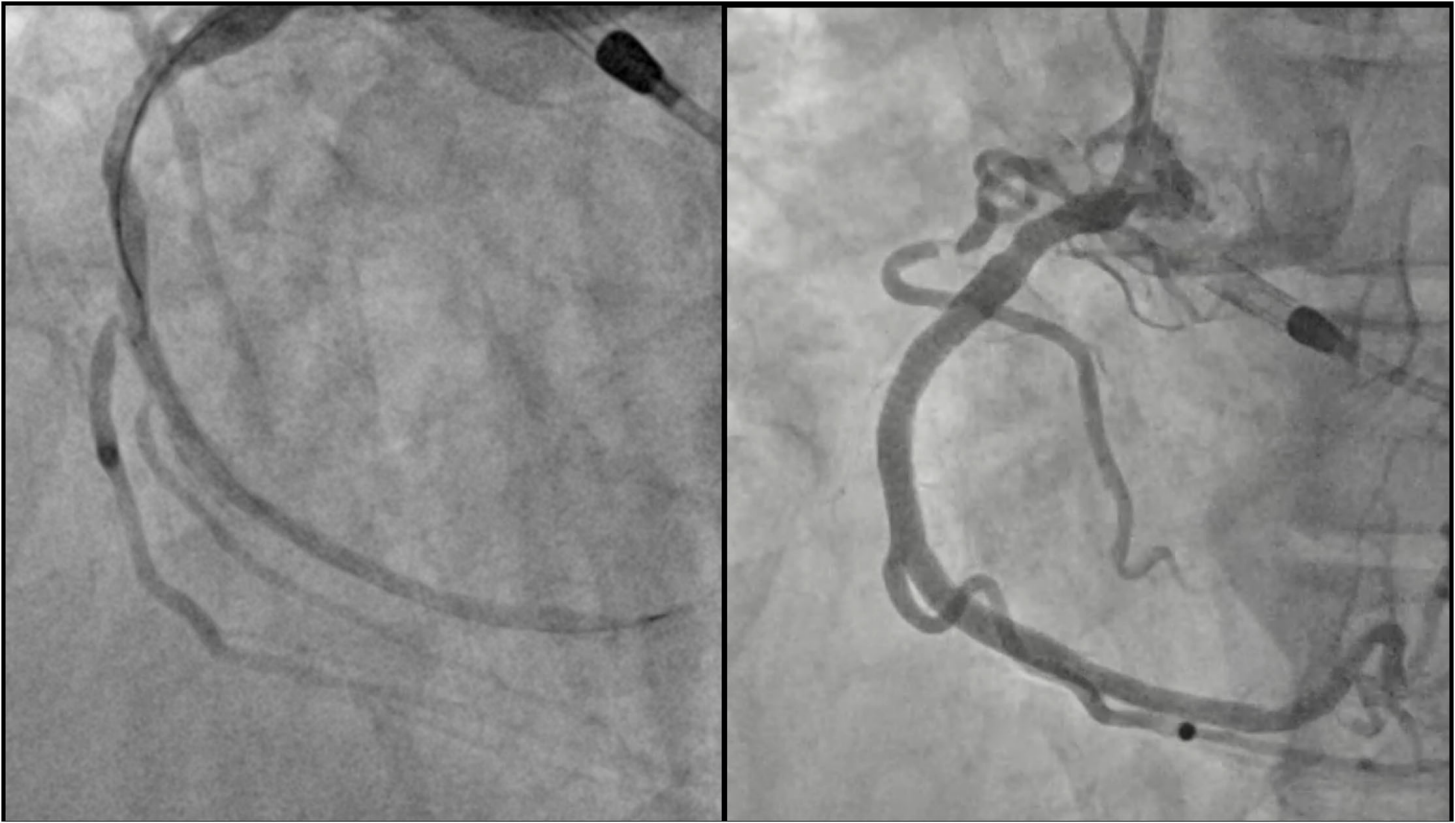
Right coronary artery after PCI. *Left panel*: result after predilatation. A severe stenosis of the marginal branch ostium is shown. Note the Supercross 120° microcatheter that was used to wire the branch. *Right panel*: final result after deployment of the Tryton side branch stent in the marginal branch and multiple drug-eluting stent implantation in the right coronary artery.

A 45-year-old male, smoker, with no previous history of cardiovascular disease presented to our emergency department for new onset of exertional dyspnoea. Echocardiography showed LV dilatation (EDV/ESV 245/180 ml) with diffuse hypokinesia, inferior wall akinesia and reduced ejection fraction (27%). Cardiac magnetic resonance (MRI) confirmed severely depressed LV function with viable myocardium and a limited subendocardial scar suggesting hibernating myocardium, potentially reversible by revascularization. Coronary angiography showed chronic total occlusions (CTOs) of the proximal right coronary artery (RCA) and mid left circumflex (LCx) coronary artery (Figure 1). The case was discussed with the heart team that decided to treat the patient with Impella-supported PCI because of the high surgical risk related to the depressed LV function and patient's preference. The right femoral access was used to introduce the Impella CP device through a 14-Fr introducer with preclosure by two suture-mediated closure devices (ProGlide, Abbott Vascular Devices, Redwood City, CA, USA). A detailed description of percutaneous catheter-based left ventricular support using the Impella CP device has been previously reported (8). A dual access (left common femoral artery with a 7-Fr introducer and right radial artery with a 6-Fr introducer) was obtained for visualization of the occluded vessels and contra-lateral coronary injection. The CTO of the LCx was successfully crossed using a Fielder XT guidewire (Asahi Intecc Co., Ltd, Japan) supported by a microcatheter (Finecross, Terumo Medical, Corp., Japan) and predilatation of the obtuse marginal branch was performed. At this point, ventricular fibrillation (VF) occurred but the patient remained conscious and hemodynamically stable. We asked the nurse to prepare the sedation before direct current (DC) shock. However, about 1 min later, spontaneous termination of the tachyarrhythmia occurred. The Impella CP parameters during the procedure and the hemodynamic support provided by the device during VF are shown in Figure 2. PCI of the LCx and obtuse marginal branch was successfully performed with implantation of two drug-eluting stents (DES) using a T-stent technique (Figure 3). Afterwards, we attempted PCI of the RCA occlusion using a dual-lumen microcatheter (Crusade, Kaneka Medical Products, Japan) to perform a parallel-wire technique. After crossing the CTO with a 0.014” Gaia Third guidewire (Asahi Intecc Co., Ltd, Japan) and predilation with a 2.0-mm semi-compliant balloon, coronary angiography showed a dominant RCA with a bifurcation lesion involving a large marginal branch. A Supercross 120° microcatheter (Teleflex Inc., Morrisville, NC, USA) was used to wire the branch and PCI was performed deploying a dedicated stent for bifurcation lesions (Tryton side branch stent, Cardinal Health Inc., Dublin, Ohio, USA) in the marginal branch and multiple DES in the main vessel with a good angiographic result (Figure 4). Of note, after the short VF episode, a stable hemodynamic status was maintained by the Impella CP support during this complex procedure. After withdrawal of the Impella and removal of the 14-Fr sheath, haemostasis was successfully obtained by tightening the two ProGlide sutures. Afterwards, the patient was treated with i.v. levosimendan. Three days later, the echocardiogram showed reduction of LV volume (EDV/ESV from 245/180 to 130/53 ml), persistent inferior akinesia and normalization of LV function (EF 59%), confirming the hypothesis of hibernating myocardium suggested by MRI. The patient was discharged 5 days after PCI. At 30-day follow-up, he was asymptomatic and resumed moderate physical activity. At 9 months, he resumed full physical activity and the echocardiogram showed an EF of 60%.

## Discussion

Patients with multivessel or left main coronary artery disease (CAD) and severely depressed LV function are generally considered for revascularization by coronary artery bypass graft surgery (CABG). Such patients may also be deemed potential candidates for high-risk PCI. In our case, due to patient's preference for PCI over CABG, the heart team decision was to proceed with a percutaneous intervention. This option, is potentially hazardous as transient ischemia caused by coronary balloon inflation and stent deployment may result in hemodynamic collapse or lethal arrhythmias. Thus, we used circulatory support with the Impella CP device, initiated prior to the intervention that allowed successful PCI of the two CTOs without abrupt cardiovascular deterioration during the procedure. Beside procedural safety, the possibility of complete revascularization during Impella-protected PCI has demonstrated a reduction of re-hospitalization (9). It is remarkable that the patient remained conscious and hemodynamically stable during VF that lasted about 1 min and spontaneously terminated, likely because Impella support maintained coronary perfusion and LV unloading. Indeed, effective LV unloading provided by the Impella pump has a clear benefit in patients with severely impaired LV function because it can effectively support the failing circulation. A growing body of registries and observational data suggests an important role for the Impella system in the treatment of selected high-risk PCI. In a systematic review of 20 studies (4 randomized controlled trials [RCTs], 2 controlled observational studies, and 14 uncontrolled observational studies) in 1,287 patients, the Impella device was found to improve procedural and hemodynamic parameters (10, 11). However, large RCTs will be needed to conclusively provide the level of clinical evidence needed to achieve a Class I guideline/recommendation for Impella support for high-risk PCI. The PROTECT IV (ClinicalTrials.gov Identifier: NCT04763200), a large, prospective, multi-center RCT is ongoing for assessing the effectiveness and safety of Impella-supported PCI compared with IABP to achieve complete revascularization and improve outcome in high-risk patients with complex CAD and reduced LV function. The trial is also supposed to clarify which patients benefit most from this approach.

## Conclusion

This case confirms the Impella pivotal role in supporting complex high-risk PCI, particularly in patients with multivessel CAD and depressed LV function even in presence of malignant tachyarrhythmias such as ventricular fibrillation.

## Author's Note

This paper was the original work of the authors who have all seen and approved of the paper and authorship. The article has not been published elsewhere and is not under consideration in any other journals.

## Data Availability Statement

The raw data supporting the conclusions of this article will be made available by the authors, without undue reservation.

## Ethics Statement

Written informed consent was obtained from the individual's legal guardian/next of kin for the publication of any potentially identifiable images or data included in this article.

## Author Contributions

GM have collected data for the paper writing and edited the figures. GM, LG, and AB performed the procedure. PO and AB contributed in the writing of the manuscript and the final revision of the manuscript.

## Funding

Funds for open access publication fees were granted by Fondazione Monzino.

## Conflict of Interest

The authors declare that the research was conducted in the absence of any commercial or financial relationships that could be construed as a potential conflict of interest.

## Publisher's Note

All claims expressed in this article are solely those of the authors and do not necessarily represent those of their affiliated organizations, or those of the publisher, the editors and the reviewers. Any product that may be evaluated in this article, or claim that may be made by its manufacturer, is not guaranteed or endorsed by the publisher.
